# Cag Type IV Secretion System: CagI Independent Bacterial Surface Localization of CagA

**DOI:** 10.1371/journal.pone.0074620

**Published:** 2013-09-10

**Authors:** Navin Kumar, Mohd Shariq, Rajesh Kumari, Rakesh K. Tyagi, Gauranga Mukhopadhyay

**Affiliations:** Special Centre for Molecular Medicine, Jawaharlal Nehru University, New Delhi, India; Centre National de la Recherche Scientifique, Aix-Marseille Université, France

## Abstract

*Helicobacter pylori* Cag type IV secretion system (Cag-T4SS) is a multi-component transporter of oncoprotein CagA across the bacterial membranes into the host epithelial cells. To understand the role of unique Cag-T4SS component CagI in CagA translocation, we have characterized it by biochemical and microscopic approaches. We observed that CagI is a predominantly membrane attached periplasmic protein partially exposed to the bacterial surface especially on the pili. The association of the protein with membrane appeared to be loose as it could be easily recovered in soluble fraction. We documented that the stability of the protein is dependent on several key components of the secretion system and it has multiple interacting partners including a non-cag-PAI protein HP1489. Translocation of CagA across the bacterial membranes to cell surface is CagI-independent process. The observations made herein are expected to assist in providing an insight into the substrate translocation by the Cag-T4SS system and *Helicobacter pylori* pathogenesis.

## Introduction

Patients with gastritis, peptic ulcer and malignancies are often infected with *Helicobacter pylori* (*H. pylori*) type I strains [1,2]. *H. pylori* acquired a cluster of genes known as *cag*-pathogenicity island (*cag*-PAI) from some unknown sources by horizontal gene transfer [3,4]. *cag*-PAI is 40 kb in size and encodes ~30 genes, which are involved in the biogenesis of a specialized macromolecular transport system called type IV secretion system (Cag-T4SS) [4]. Cag-T4SS in *H. pylori* is responsible for translocation of cytotoxin associated antigen A (CagA), peptidoglycan and possibly other unidentified factors across the bacterial cell envelopes into human gastric epithelial cells leading to pathogenesis [4–7].

T4SSs are ancestrally related to the bacterial conjugation systems and a number of pathogenic Gram-negative bacteria utilize it either to acquire genetic materials or transport proteins (toxin) or DNA-protein complexes into surrounding environment or into host [8,9].

One of the best studied prototypes of T4SS is the VirB/VirD4 system of *Agrobacterium tumefaciens* (*A. tumefaciens*) [10,11]. Recent studies have, however, reported marked differences between *cag*-PAI encoded T4SS (Cag-T4SS) and other known T4SS in composition and substrates translocation mechanism [12,13]. Several components of Cag-T4SS are unique and have no significant similarity (based on gene sequence and topological analysis) with components of other bacterial T4SSs like *A. tumefaciens* [11].

One such unique *cag*-PAI protein is CagI (HP0540) which is essential for CagA translocation but is not required for IL-8 induction in human gastric epithelial cells [7]. Recent studies, however, have reported involvement of the protein in IL-8 induction contradicting the earlier result [14]. CagI has no sequence homology with any other components of T4SS in bacterial species. Following bioinformatics analysis it has been predicted that CagI has N-terminal hydrophobic helix spanning residues 26-51 for inner membrane localization and presumed to play an essential role as a carrier protein in recruiting CagA into Cag-T4SS on its cytosolic face [11]. On the contrary, Jimenez-Soto et al., proposed that host β1 integrin acting as a cellular receptor for Cag-T4SS interacts directly with Cag-T4SS components CagY, CagA and CagI. Moreover, they predicted that these subunits of Cag-T4SS should be accessible on the surface of the Cag-T4SS pilus for β1 integrin binding. Subsequently, they verified experimentally that CagY and CagA are located on the surface of the Cag-T4SS pilus by field scanning electron microscopy (FESEM) [13]. However, they did not provide any experimental evidence for CagI localization in *H. pylori*.

In the prototypic model system of T4SS in *A. tumefaciens* VirB2 and VirB5 are reported to be pilus associated proteins [15]. Both VirB2 and VirB5 are also detected in the periplasmic fraction of *A. tumefaciens*. Interestingly, *H. pylori cag*-PAI protein CagC (HP0546) is predicted to possess sequence and structural similarities to VirB2-like pilins of *A. tumefaciens* that is localized on the surface appendages of *H. pylori* [16]. CagY (HP0527), a VirB10 homologue, has been shown to be surface associated [17]. In another proposed model it was suggested that the Cag-T4SS pilus is decorated locally or entirely by CagY [17–19]. CagX, a VirB9 homologue, is a surface exposed protein and speculated that it forms a secretin-like pore in the outer membrane of *H. pylori* that may allow passage of the pilus and substrate across the outer membrane [20]. CagT, a VirB7 homologue, is located at the pilus site and proposed that it might form oligomeric ring like-structure around the base of the pilus assembly [17]. Kutter et al., 2008 observed mutual interactions between CagY, CagX, CagM, and CagT by immunoprecipitation (IP). It was suggested that CagY, CagX, CagT, and CagM form the core channel complex at the periplasmic face of the bacterium.

Immunoelectron microscopy revealed that *H. pylori* consist of a pilus structure protruding from the cell surface on the wild-type but not on *cag*-PAI deleted *H. pylori* strain. Induction of Cag-T4SS pilus is reported to be contact dependent [17,21,22]. Notably, β1 integrin acts as a host cell surface receptor for the *cag*-PAI secretion system and essential for translocation of CagA. Cag-T4SS pilus component CagL interacts with α5β1 integrin via its arginine-glycine-aspartate (RGD) motif and essential for CagA translocation into host cell [21]. But subsequently in a conflicting report it was claimed that RGD motif is not required for interaction between *H. pylori* and α5β1 integrin [13]. In addition to this, CagA, CagY and CagI are also found to interact directly with β1 integrin. Based on this, the authors predicted that these subunits of Cag-T4SS should be accessible on the surface of the Cag-T4SS pilus for β1 integrin binding. However, biogenesis of the Cag-T4SS pilus structure and its role in CagA translocation mechanism are not well defined as yet.

Scanning electron microscopy revealed that CagI, CagH and CagL play key roles in Cag-T4SS pilus formation at the bacteria-host interface and CagH regulates the size of the pilus during infection [22]. CagI co-transcribe along with CagL and CagH and forms a complex with CagL [14,22]. Moreover, stability of CagI is impaired in the absence of several important *cag*-PAI genes suggesting that intact secretion system is essential for CagI stability [14]. However, it is not well characterized whether CagI is pilus component or interacts with CagH on the pilus surface. In silico analysis revealed interaction of CagI with HP1489, a non-cag-PAI protein [23]. HP1489 is identified as a TolC homologue, an outer membrane efflux protein of multi-drug efflux transporter system, involved in drug efflux [24].

In the present study, following biochemical, fluorescence and electron microscopic analyses, we have characterized a unique component of Cag-T4SS, CagI and decipher its role in CagA translocation across the bacterial membranes. Previous studies have shown conflicting results about CagI localization in *H. pylori*. Two studies found that CagI is present exclusively in the membrane [22,25] while the other reported its presence in both soluble and membrane pools [14]. In this study we provide evidences that CagI is distributed in the periplasmic space towards the perimeter of outer membrane (loosely attached to the membrane) as well as exposed to the bacterial cell surface particularly on the pili. CagI forms complex with CagH that is independent of other Cag-T4SS components. In addition we have also analyzed the expression and stability of the protein in isogenic *Hp*Δ*cagZ*, *Hp*Δ*cagY*, *Hp*Δ*cagX*, *Hp*Δ*cagV*, *Hp*Δ*cagT*, *Hp*Δ*cagM*, *Hp*Δ*cagA*, *Hp*Δ*cagδ*, *Hp*Δ*cagH*, *Hp*Δ*cagG*, and *Hp*Δ*cagE* mutants of *H. pylori* strain 26695 and discussed its role in CagA translocation.

## Results

### Localization of CagI

**Figure 1:**
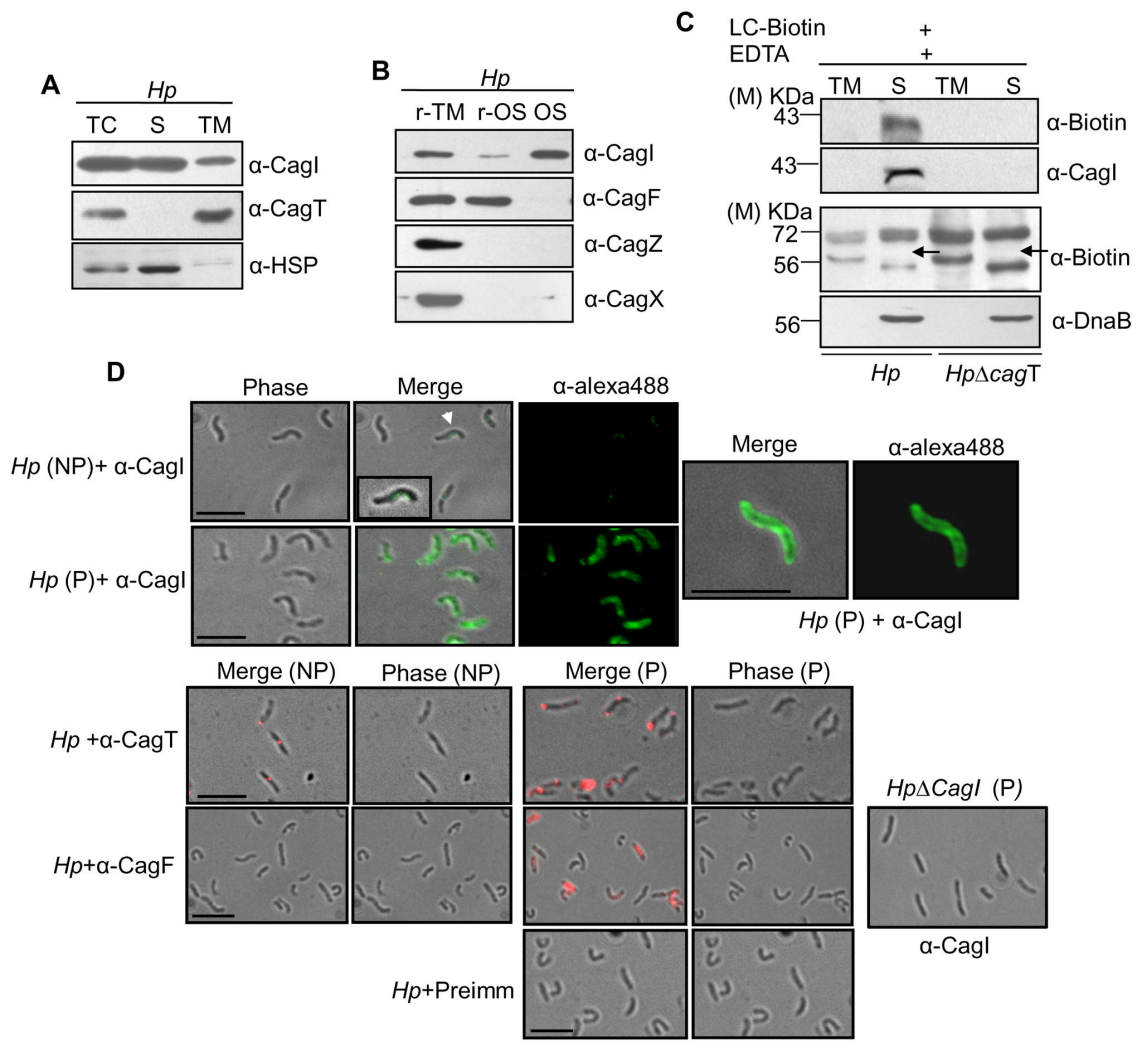
Cellular localization of CagI in *H. pylori*. (**A**) Western blots showing sub-cellular fractionation of wild-type *H. pylori*. TC, S and TM indicate total-cell lysate, soluble (cytoplasmic/periplasmic) and total membrane fractions respectively. Volume of TM was adjusted corresponding to that of S fraction and then equal volume of each was loaded in to gel. (**B**) Western blots showing osmotic shock analysis of *H. pylori*. r-TM, r-OS, and Os stand for residual total membrane, residual osmotic shocked content (cytosolic contents) and osmotic shocked fraction (periplasmic contents) respectively. Antibodies used are marked. (**C**) Western blots showing selective biotinylation of CagI from biotin labeled wild-type *H. pylori* (Hp) and HpΔcagT mutant strains. TM and S stand for total membrane fraction and soluble fraction. Antibodies used are marked. M-indicates molecular size standard. Arrow indicates position of non-biotinylated DnaB. (**D**) Immunofluorescence microscopy showing cellular localization of CagI in wild-type *H. pylori* (Hp) under permeabilized (P) and non-permeabilized (NP) conditions. *H. pylori* cells were fixed and permeabilized with 0.2% Triton X-100 and probed with antibodies as indicated. Alexa fluor 488 (green colour) and Alexa fluor 594 (red colour) conjugated secondary antibodies were used for signal generation and finally examined by immunofluorescence microscopy. Permeabilized *HpΔcagI* cells are showing specificity of α-CagI antibody. Pre-immune serum was used as control antibody. Scale bars indicate 5µM.

To characterize CagI, one of the unique *cag*-PAI components, we first examined its cellular localization. We fractionated wild-type *H. pylori* cells (26695) into soluble cytoplasmic/periplasmic and total membrane fractions and tested the presence of CagI by Western blotting using anti-CagI antibody. As shown in Figure **1A**, unlike control protein CagT, CagI was recovered mostly in the soluble cytoplasmic/periplasmic fraction and to a lesser extent in the membrane fraction. Outer membrane-associated CagT was tested as a marker protein for membrane fraction and HSP (Santa Cruz) was tested for soluble protein. To identify the source of the protein in the soluble fraction we subjected freshly grown wild-type *H. pylori* cells to osmotic shock and analyzed the resultant supernatant for the presence of CagI. CagI was detected exclusively in the osmotic shocked fraction (Figure **1B**). As control CagF, CagZ, and CagX were tested for cytosolic, inner membrane and outer membrane proteins respectively. Selective biotinylation analysis of the periplasmic proteins in *H. pylori* also identified CagI in the periplasm (Figure **1C**) [26]. To gain further insight, immunofluorescence microscopy (IFM) was performed on wild-type *H. pylori* cells under permeabilized and non-permeabilized conditions using anti-CagI, anti-CagT and anti-CagF antibodies. CagT and CagF were tested as marker for surface and inner membrane-associated proteins respectively. As shown in Figure **1D**, under permeabilized condition CagI signals were detected at multiple foci along the perimeter of the bacterium that may be partially associated with the outer membrane. No other tested *cag*-PAI components showed similar kind of fluorescence distribution pattern. Interestingly, in a recent report a similar pattern is being reported for membrane and periplasmic components of *A. tumefaciens* T4SS by immunofluorescence deconvolution microscopy [27,28]. Under non-permeabilized condition only CagT and CagI signals were detected on the cell surface as foci (albeit CagI signals were rather weak). No CagF specific signal was, however, detected under non-permeabilized condition as expected. Taken together, these results suggest that CagI may be periplasmic protein loosely associated with the outer membrane and a few of them partly exposed on the bacterial surface.

### CagI is pili associated component of Cag-T4SS

**Figure 2:**
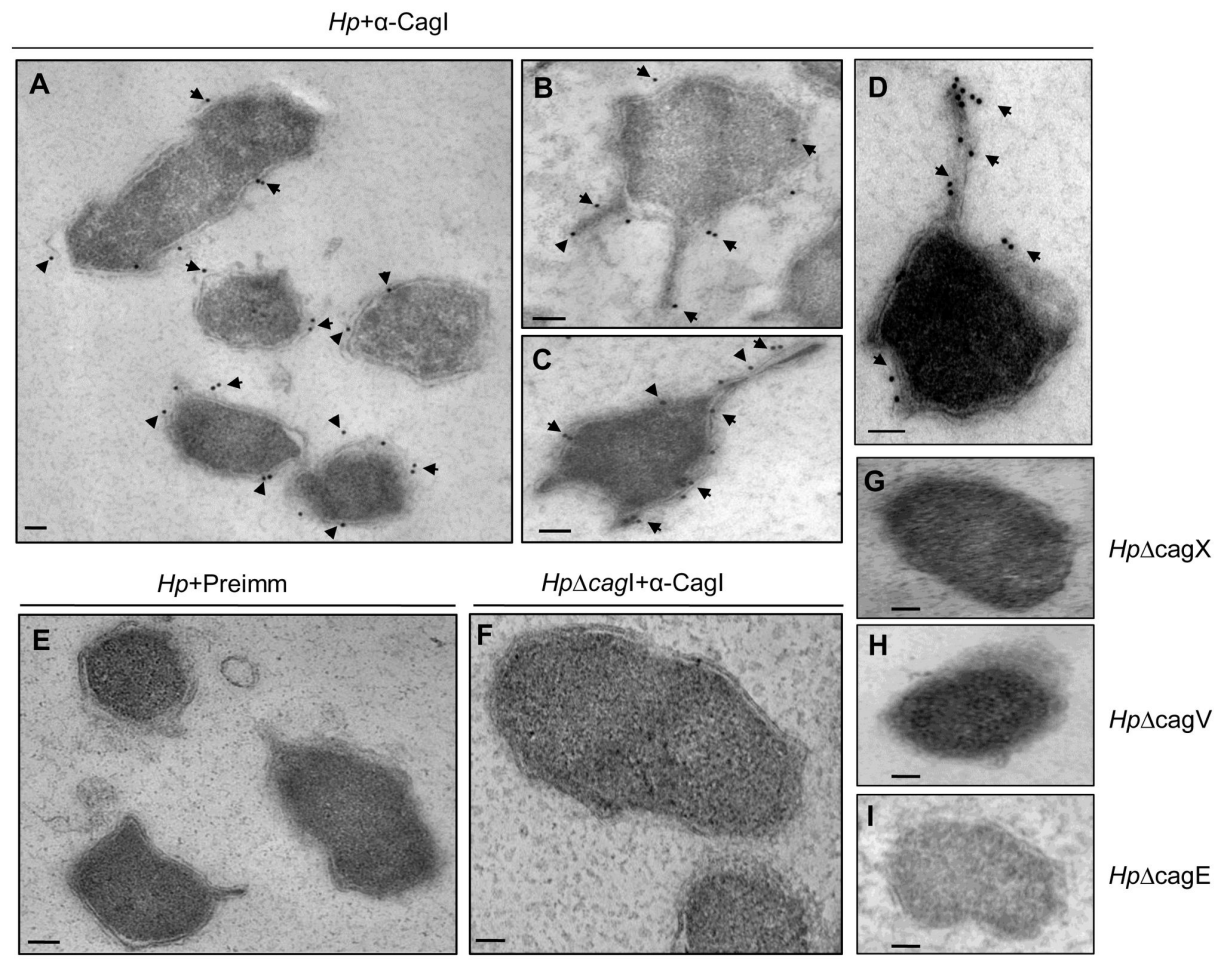
Immunogold electron microscopy (IEM) reveals pili like structure associated CagI in wild-type *H. pylori (Hp).* *H. pylori* strains were grown on BHI agar plates and immunogold labeling of ultrathin sections was performed as described in Materials and Methods. (**A**, **B**, **C**, and **D**) Wild-type *H. pylori* stained with anti-CagI and gold-labeled secondary antibody. (**E**) Wild- type *H. pylori* stained with rabbit pre-immune (preimm.) serum and gold-labeled secondary antibody. (**F**) Isogeneic mutant *Hp∆cagI* stained with anti-CagI and gold-labeled secondary antibody. (**G**, **H**, and **I**) Isogenic Hp∆cagX, Hp∆cagV, and Hp∆cagE mutant strains showing absence of pili in TEM sections. Scale bars indicate 100 nm. Arrowheads indicate location of gold-labeled secondary antibody.

In two recent reports using recombinant proteins in co-culture experiment and complementation analysis respectively, CagI has been shown to be a cell surface exposed protein which is required for *H. pylori* pili formation [14,22]. Following a cue from our initial experimental data of CagI localization we performed transmission electron microscopic (TEM) analysis of wild-type *H. pylori* and its isogenic mutants grown in BHI serum media to study direct involvement of native CagI in pili formation. In agreement with our IFM results we observed CagI specific gold spots along the outer membrane on the wild-type bacterial surface and in some cases in the periplasmic space (Figures **2A, 2B**, **2C**, and **2D**). These signals were absent in *Hp*Δ*cagI* isogenic *H. pylori* mutant strain and in wild-type bacterium where control IgG was used (Figures **2E**, and **2F**). Surprisingly, in some of the ultrathin sections of wild-type bacteria only, we observed unusual surface structures (Figure **2B, 2C** **, and 2D**). Number of such structures per section varied between 1–3. We observed relatively more CagI specific gold spots on those structures compared to rest of the cell surface (Figure **2B, 2C** **, and 2D**). When we repeated TEM on mutant strains of *H. pylori* lacking key Cag-T4SS genes, like isogenic *Hp*Δ*cagX*, *Hp*Δ*cagV*, and *Hp*Δ*cagE* mutant strains, we observed no such structure in hundreds of ultrathin sections tested (Figure **2G, 2H** **, and 2I**). We also didn’t detect the structure in *HpΔcag*I strain (Figure **2F**). All these experiments were performed in the absence of host cell. It is important to note that Rohde et al., 2003 and Tanaka et al., 2003, also reported such type of surface structures in wild-type *H. pylori* strain 26695 and they called these ‘surface appendages’ and ‘appendices’ respectively [17,20]. Therefore to ascertain the nature of the surface structure we conducted SEM on co-cultured wild-type *H. pylori* and its isogenic mutants for example *Hp*Δ*cagI, Hp*Δ*cagV, Hp*Δ*cagT*, and *Hp*Δ*cagδ*. Interestingly, we observed comparable results, only so called ‘surface structures’ were replaced by pili in wild-type bacteria as demonstrated in a recent publication [22]. No pilus was however, detected in the mutant strains grown in co-culture (**Figure S5 **in File **S1**). Therefore, we considered the surface structure observed in TEM as pili. It is important to note that in previous studies no association of native CagI with pili was experimentally demonstrated in *H. pylori* in the absence of host cells. In summary, we provide experimental evidence of pili formation in wild-type *H. pylori* under non-infection condition. We also report presence of native CagI on the pili surface. We speculate that multiple CagI foci detected in permeabilized bacteria could be potential hot spot for biogenesis of multiple Cag-T4SS or pili to maximize infectivity.

### CagI has multiple interacting partners including non-cag-PAI component HP1489

**Figure 3:**
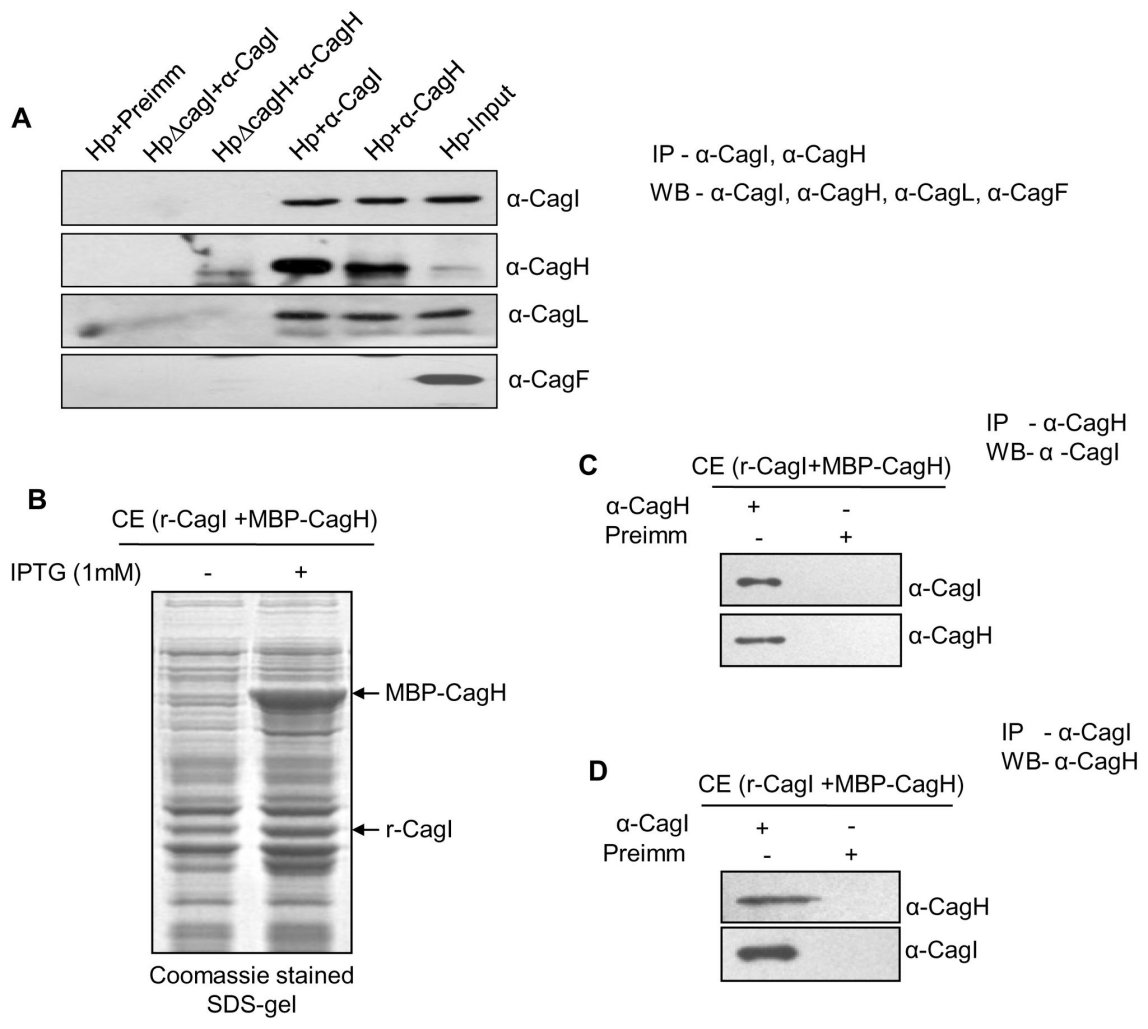
Co-immunoprecipitation (Co-IP) showing interaction between native CagI and CagH in *H. pylori*(Hp). (**A**) Western blots showing Co-IP of CagH and CagL by anti-CagI and Co-IP of CagI and CagL by anti-CagH antibody from *H. pylori* cell extract. (**B**) SDS-PAGE showing co-expression of MBP-tagged CagH and without tag CagI. (**C** and **D**) Western blots showing Co-IP of recombinant CagI (without tag) and MBP-CagH by anti-CagH and anti-CagI antibodies respectively. Antibodies used in Western blots are indicated.

**Figure 4:**
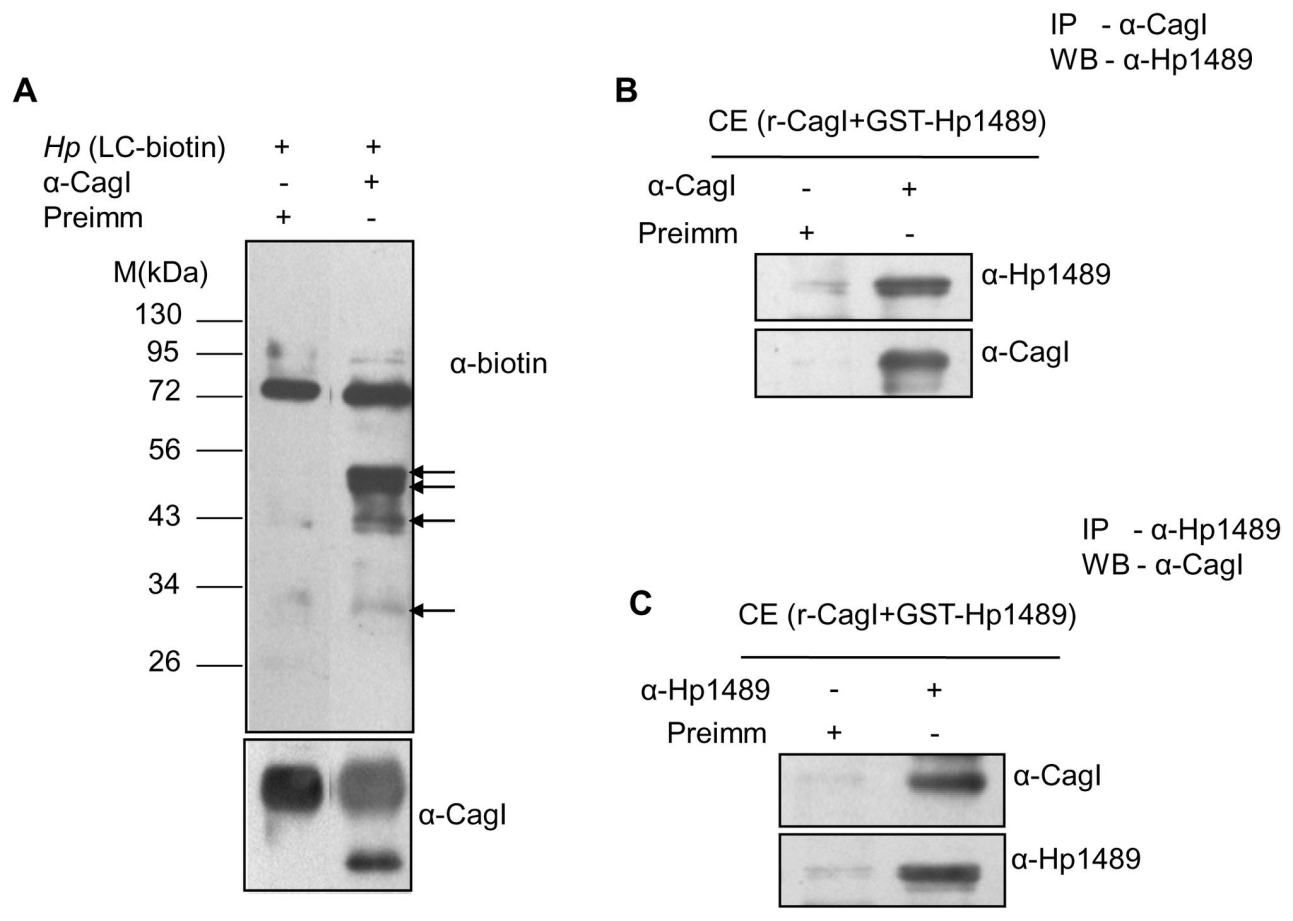
Co-immunoprecipitation (Co-IP) from biotinylated *H. pylori* extract and recombinant proteins co-expressed in *E. coli* showing interacting partners of CagI. (**A**) Western blots showing co-immunoprecipitation of biotinylated proteins by anti-CagI antibody from biotin labeled *H. pylori* extract. Blots were probed with HRP-conjugated anti-biotin antibody (α-biotin) and anti-CagI antibody (α-CagI). Arrows indicate biotinylated protein bands. (**B** and **C**) Western blots showing Co-IP of recombinant Hp1489 (GST-tag) and CagI (without tag) by anti-CagI and anti-Hp1489 antibodies respectively. Antibodies used in Western blots are indicated. M-indicates standard protein size markers.

Recently, CagI has been shown to interact with CagL, CagH and host factor β1 integrin [13,14,22]. Among these both CagL and CagH are encoded by the same operon that codes for CagI. CagA on the contrary is the substrate of the Cag-T4SS that requires CagI for its translocation across the cell envelope into the host cytoplasm [22]. To reassess interaction between CagI and the *H. pylori* components we performed immunoprecipitation from soluble extract prepared from wild-type *H. pylori* and isogenic mutant strains, and *E. coli* co-expressing recombinant CagI and the putative interacting proteins. In agreement with the published CagI interaction results we observed co-immunoprecipitation of native CagI and CagL by anti-CagH antibody and native CagL and CagH by anti-CagI antibody from cell extract prepared from wild-type strain (Figure **3A**) [14,22]. CagF was used as a negative control. We observed direct interaction between recombinant CagI and CagH proteins co-expressed in *E. coli* (Figure **3B, 3C** **, and 3D**). Since CagI was detected mostly in the soluble periplasmic fraction and to a limited extent in the membrane fraction we thought it would be practical to look for other CagI interacting proteins, if any, in the periplasmic space. Previously, at least two non-*cag*-PAI components HP1489 and GyrA have been predicted to interact with CagI by *in-silico* analysis [23,29]. In this direction, we have selectively labeled periplasmic proteins in wild-type *H. pylori* by biotin following published procedure and extract prepared from it was used in immunoprecipitation experiment using anti-CagI antibody [26]. As shown in Figure **4A**, at least 3 biotin-labeled proteins were co-immunoprecipitated by anti-CagI antibody. Two of the first migrating proteins were later identified as CagL and CagH by immunoprecipitation and Western blotting. The slow migrating biotin-labeled protein was expected to be non-*cag*-PAI component HP1489 as predicted by *in-silico* analysis. In the next step the gene for HP1489 was cloned and the recombinant protein was co-expressed with CagI in *E. coli* and soluble proteins were tested for interaction by immunoprecipitation. Results of the experiments are shown in Figure **4B** **, and 4C**. Unlike individually expressed recombinant proteins in *E. coli*, co-expressed proteins are recovered in soluble fraction as interacting partners.

### CagI is unstable in isogenic mutants of *cag*-PAI

Several studies have shown that stability of one or more component(s) in multi-protein complexes dependent upon presence of other component(s) and are well-documented in the prototypical T4SS in *A. tumefaciens* [30]. Likewise, in *H. pylori*, it was observed that the quantity of CagT was significantly reduced in *Hp*Δ*cagX*, and *Hp*Δ*cagM* mutants in contrast to wild-type *H. pylori* strain [18]. Similarly, Cagδ and CagT were also found to mutually stabilize each other [31]. Therefore, we tested the stability of CagI and CagH in the background of various deletion mutants of *cag*-PAI genes, essential for CagA translocation. In order to do this, different isogenic deletion mutants of *H. pylori* were grown in BHI medium supplemented with required antibiotics and respective extract were prepared. Next, equal amount of extract from individual mutant strains were resolved in SDS-PAGE and subjected to Western blotting using anti-CagI, anti-CagH, anti-CagA and anti-OMP antibodies.

**Figure 5:**
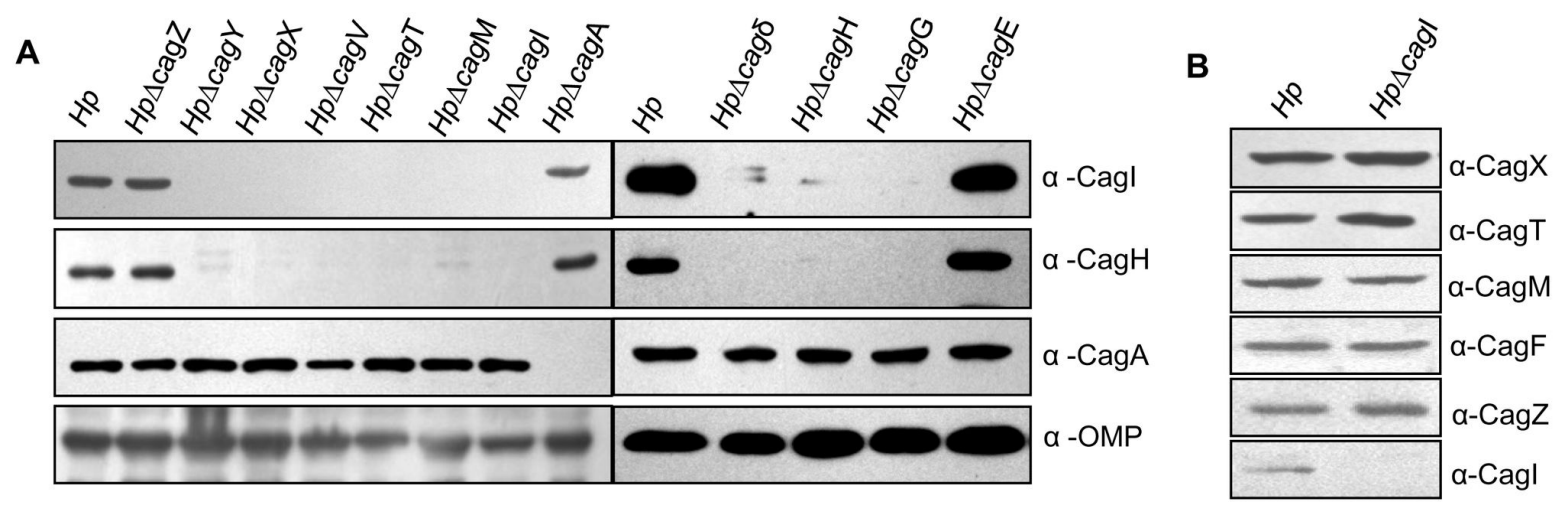
Western blots showing expression of CagI and CagH in mutant *H. pylori* strains (cag-PAI genes) in comparison to wild-type *H. pylori*(Hp). (**A**) Equal amounts (OD600-2.0) of all indicated Hp cells were lysed in SDS sample buffer and were separated in SDS-PAGE, transferred to PVDF membrane and Western blotted. (**B**) Stabilities of Cag-T4SS components CagX, CagT, CagM, CagF, and CagZ in isogenic *HpΔcagI* mutant strain. Antibodies used are indicated. OMP was used as loading control.

As shown in Figure **5A**, both CagI and CagH were not detected in *Hp*Δ*cagY, Hp*Δ*cagX, Hp*Δ*cagV, Hp*Δ*cagT, Hp*Δ*cagM, Hp*Δ*cagδ, and Hp*Δ*cagG* isogenic mutants of *H. pylori* 26695 strain. Likewise, CagH was also not detected in *Hp*Δ*cagI* mutant strain. On the contrary, both CagI and CagH were produced at the wild-type levels in isogenic *Hp*Δ*cagZ, Hp*Δ*cagA* and *Hp*Δ*cagE* mutants of *H. pylori*. CagA and OMP were, however, detected in significant amount in all the mutant strains tested. Level of expressed OMP was tested as a loading control. Absence of CagH in the background of *cagI* deletion suggests that the stability of CagH is CagI dependent. Overall presence of productive Cag-T4SS is required for CagI and CagH stability. However, stabilities of key Cag-T4SS components like CagX, CagT, CagM, CagF, and CagZ remain unaffected in the absence of CagI (Figure **5B**).

### CagI independent surface localization of CagA

**Figure 6:**
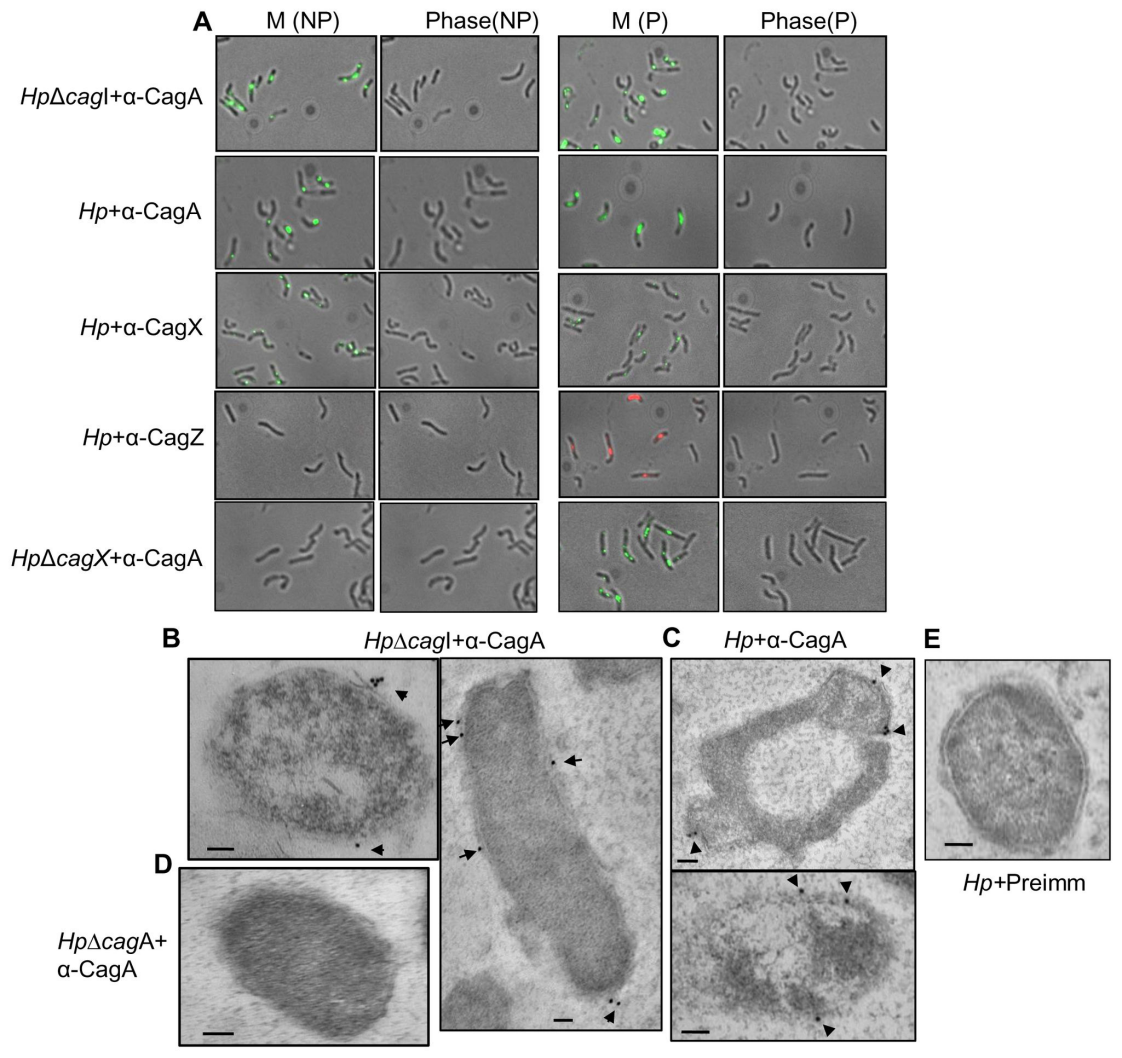
Surface localization of CagA in wild-type and *HpΔcagI* strains. (**A**) **Immunofluorescence microscopy showing surface localization of CagA in wild-type and *HpΔcagI* strains**. Wild-type and *HpΔcagI* cells were fixed and one set was permeabilized with 0.2% Triton X-100. M, NP and P stand for merge, non-permeabilized and permeabilized cell respectively. Primary antibodies used in IFM are indicated. Secondary antibodies used were Alexa fluor 488 (green colour) and Alexa fluor 594 (red colour) conjugated. (**B** and **C**) **Immunogold electron microscopy (IEM) showing surface localization of CagA in *Hp*Δ*cagI* and wild-type *H. pylori* (Hp) strains**. Immunogold labeling of *H. pylori* strains ultrathin sections were performed as described in Materials and Methods. (**B**) *HpΔcagI* cells were stained with anti-CagA and gold-labeled secondary antibody. (**C**) Wild-type *H. pylori* cells were stained with anti-CagA and gold-labeled secondary antibody. (**D**) *HpΔcagA* cells were stained with anti-CagA and gold-labeled secondary antibody. (**E**) Pre-immune (preimm.) serum was used as negative control. Scale bars indicate 100 nm. Arrowheads indicate location of gold-labeled secondary antibody.

While studying surface localization of Cag components, we tested CagA, the substrate of Cag-T4SS, CagX (positive control) and CagZ (negative control) by IFM. CagX is reported earlier as partially surface exposed outer membrane associated protein and tested as positive control [17,20]. However, CagZ was tested as negative control since it is known as an inner membrane associated protein [32]. We used permeabilized and non-permeabilized wild-type *H. pylori* and isogenic *Hp∆cagI* strain grown on BHI-serum agar plate for IFM. As shown in Figure **6A**, like CagX, CagA was detected on the bacterial surface under non-permeabilized condition. As expected, no CagZ signal was observed under similar condition. The results were confirmed multiple times on non-permeabilized wild-type cells. These observations suggested that CagA could be a cell surface exposed protein as predicted earlier [21]. To substantiate the results, we performed TEM on wild-type and *Hp*Δ*cagI H. pylori* strains. As shown in Figure **6B and 6C**, CagA was again observed on the bacterial cell surface of the sections tested. However, no CagA specific signal was detected in negative control *Hp*Δ*cagA* and in wild-type strain where pre-immune serum was used (Figures **6D**, and **6E**). It is important to mention that earlier CagA and CagT were observed co-localized near the bacteria-host cell interface under infection condition [20]. This is the first experimental evidence of CagA surface localization in the absence of host cell contact. This result raised an obvious question regarding CagA translocation, especially across the bacterial membranes. Previous and recent studies have identified several conserved and unique components of Cag-T4SS that are required for CagA translocation [7,14,22]. Among these, CagT, CagX, CagY, and CagV are conserved Cag-T4SS structural components. CagI, CagH and CagL are on the other hand unique to Cag-T4SS. Recently, CagI is identified as surface-associated protein along with CagL and CagH [22]. Therefore, to have an insight into the CagA translocation and role of CagI in the process, we repeated IFM and TEM on isogenic *Hp∆cagI H. pylori* strain. To our surprise we detected CagA on the bacterial surface even in the absence of the protein (Figures **6A**, and **6B**). As expected no surface associated CagA signal was detected in the absence of Cag-T4SS structural component CagX (Figure **6A**). Subsequently, absence of CagI does not affect the presence of CagA in membrane fraction (**Figure S3 **in File **S1**). Taken together, these results suggested that CagA may not require CagI for its translocation across the bacterial membranes but essential in further translocation into the host cell. We hypothesize that there could be two steps process that leads to CagA translocation from bacterial cytoplasm into host cell.

## Discussion

*H. pylori* are one of the most successful human pathogens. Since its discovery as the causative agent of gastritis and other pathological conditions a number of studies have been undertaken to understand its epidemiology, genetics and pathology [7,33–35]. Over the years we have acquired knowledge regarding the bacterium’s survival in the extreme acidic environment in the human lumen, modulation of host cellular signaling and evasion of host defense mechanism [33]. Earlier, sequence analysis and subsequent confirmatory studies identified two functional T4SS; one for transport of cytotoxin CagA into host gastric epithelial cells called Cag-T4SS and the other one for acquiring foreign genetic materials from unknown sources termed as Comb system [36]. Cag-T4SS is a multi-protein specialized secretion system encoded by a 40 kb DNA segment acquired by the bacterium via horizontal gene transfer system. It is a trans-envelope protein complex comprising of more than 30 proteins [7]. Some of these are homologues of components of prototypical T4SS encoded by Ti plasmid of *A. tumefaciens* and ancestrally related to bacterial conjugation systems [31]. Cag-T4SS has inner membrane associated core proteins complex comprising of CagV, Cagβ, Cagα, and CagE that are homologous to components of VirB/VirD4 system and unique factors CagZ, CagF etc. On the other hand, outer membrane core complex consists of Cagδ, CagY, CagX, CagT and CagM. Among these Cagδ and CagM are unique to Cag-T4SS. Other than these components like CagI, CagH, CagL, CagG, CagW, and CagU play roles in substrate translocation. Interestingly, most of these unique factors have no known homologues in literature and their functions except that of CagL are not clear yet. Recent studies have indicated that CagI, CagH and CagL are surface exposed components of the Cag-T4SS and might be required for or involved in pili formation [22]. Although evidence for direct involvement of the native proteins could not be experimentally demonstrated, indirect studies using tagged recombinant proteins are indicated to be required for the Cag-T4SS pili formation [22].

In the present study, we have characterized one of these proteins CagI and provide direct experimental evidence for its association with Cag-T4SS pili. Furthermore, we have shown for the first time that CagI is not involved in CagA translocation from cytoplasm to bacterial cell surface rather it may be required for facilitating its further movement to host epithelial cells (**Figure S4 **in File **S1**).

In a recent paper, Shaffer et al., reported surface association of CagI and CagH using epitope-tagged recombinant Cag proteins. They have tested the proteins by proteinase K (protease sensitivity), flow cytometry, cell fractionation studies and immunogold transmission electron microscopy (TEM). Under their study conditions they mainly found fewer than 10 CagI and CagH molecules on the cell surface. While during cell fractionation studies they obtained CagI completely in the membrane fraction. However, Pham et al., reported that CagI exists in multiple pools of membrane and soluble fractions [14]. In our study we have clearly shown localization of CagI on the cell surface (under non-permeabilized condition) and all along the inner periphery of the outer membrane in the periplasmic space (under permeabilized condition) by IFM. We corroborated our findings by cell fractionation studies, where we observed CagI in the soluble periplasmic fraction and to a lesser extent in the membrane fraction.

By TEM we showed similar localization pattern in the periplasmic space and a fraction of CagI even detectable on the cell surface primarily on the pili. It is worth to mention that CagI specific spots were relatively more on the pili. Pili have been observed under non-infection condition (pure culture) as reported earlier by Rohde et al., 2003 and Tanaka et al., 2003, although they termed those as surface appendages and appendices respectively. The width of the surface structure observed in TEM in the present study was approximately 70 nm; Rohde et al., also observed similar width for the surface appendages they reported [17,20]. It is also to mention that fractions of *H. pylori* 26695 cells grown under the experimental condition having pili structure were less than 5%. Whereas Rohde et al., 2003, reported surface appendages in about 20% of the cells grown under pure culture. These differences could be due to different growth medium used or could be an under estimation (present study) or over estimation. The average length and width of the pili we observed in wild-type *H. pylori* 26695 strain grown in co-culture by SEM were 313 nm and 62 nm respectively. In co-culture experiments also we observed less number of pili (**Figure S5 **in File **S1**). Again the difference could be due to experimental conditions used.

Regarding CagI interacting partners, we observed its interaction with CagH, CagL, β1-integrin, and also with a non-*cag*-PAI protein HP1489 earlier predicted to be an interacting partner of CagI [23]. We have shown these interactions using native proteins by immunoprecipitation experiment except for HP1489. However, direct interactions of CagI with CagH, and HP1489 were demonstrated using recombinant proteins co-expressed in *E. coli* by immunoprecipitation. However, we were unable to show physiological function of HP1489 in CagI-mediated CagA translocation in *H. pylori* due to non-availability of isogenic *Hp*Δ1489 mutant strain. As mentioned above, one of the important finding of our present study was CagI independent surface localization of CagA. Previously, we observed that in the absence of inner membrane associated components CagV and CagE and outer membrane core complex components CagY, CagX, CagT and CagM, CagA cannot reach bacterial cell surface (Figure **6A** **, and Figure S6 **in File **S1**, unpublished observation). However, we do not have any clue regarding how CagA translocate from cytoplasm to bacterial cell surface in the absence of CagI. It is interesting to note that in the absence of CagV, CagX, CagY, CagT, CagM, CagG, Cagδ, and CagH the key Cag-T4SS components, CagI is also absent and no pili structure was observed in isogenic *Hp*Δ*cagV*, *Hp*Δ*cagX*, *Hp*Δ*cagT*, *Hp*Δ*cagG*, *Hp*Δ*cagδ*, and *Hp*Δ*cagE* strains (Figure **2G, 2H** **, and 2I, and Figure S5 **in File **S1**). Therefore, it appears that intact or productive Cag-T4SS could be required for CagA surface localization. There are a number of reports regarding possible mechanism of CagA transport through Cag-T4SS including probable function of CagI [13,14,22].

Additionally, CagA and CagI have been reported to be bacterial cell surface exposed proteins that interact with host cells receptor protein β1 integrin [13,22]. It appears that CagA translocation right from cytoplasm into host epithelial cells could be a two-step process. Our report and compilation of all these published information might lead to revelation of an alternative mechanism of CagA translocation into host epithelial cells.

## Materials and Methods

### Bacterial strains and growth conditions

*H. pylori* 26695 was grown on 3.7% w/v brain heart infusion (BHI) agar (Difco) supplemented with 7% fetal calf serum (FCS), 0.4% campylobacter growth supplement and *H. pylori* dent supplement (Oxoid). Culture plates were incubated at 37°C for 24-36 h in a GasPak anaerobic system using GasPak EZ sachet (BBL). *H. pylori* isogenic mutant strains were selected on BHI-serum plates supplemented with chloramphenicol (6µg/ml). The *H. pylori* strains were maintained as frozen stocks at -70°C in 70% brain heart infusion supplemented with 20% glycerol and 10% FCS. *E. coli* strains DH5α, and BL-21 (DE3) were grown in Luria Broth liquid medium (LB) or on LB agar plates supplemented with ampicillin (100µg/ml), kanamycin (50µg/ml), chloramphenicol (30µg/ml) for the amplification of plasmid DNA and over-expression of recombinant proteins as appropriate. *H. pylori* wild-type, mutant strains and *E. coli* strains used in the present study are given in **Table S1** in File **S1**.

### Cloning-expression of genes

Plasmid DNA (vector), pET-28a was used to express CagI (without tag) and CagH (His-tag) for generation of polyclonal antibody. Plasmid pACYC-duet1 was used to clone *cagI* for co-expression with *cagH* tagged with MBP in the plasmid pMal-c-2x. For co-expression of recombinant proteins, the published protocol with minor modifications was followed [37]. Plasmid vector pACYC-Duet1 is compatible with pGEX-6p2 and pMAL-c-2x. Transformation competent BL-21(DE3) cells were transformed with pACYC-Δ32N*cagI*/pMAL-*cagH* and pACYC-Δ32N*cagI*/pGST-*hp1489* plasmid pairs separately. Primer pairs and their sequences are mentioned in **Table S2** in File **S1** and Table **1**. Transformed *E. coli* cells were selected on chloramphenicol/ampicillin (pACYC/pMAL/or pGEX) double antibiotic containing LB-agar plate. Protocol for co-expression was same as expression of single gene except culture media contained double antibiotics instead of single antibiotic.

**Table 1:** Oligonucleotides used in the present study.

fΔ32N*cagI* NcoI	5’-CATGCCATGGGAGCCATTCAGGCGGACGCAC-3’
r*cagI* HindIII	5’-CCCAAGCTTTCATTTGACAATAACTTTAGA-3’
f*cagH* BamH1	5’-CCGGATCCCCGCAAATGACCGCTATCATG-3’
f*Hp1489*BamHI	5’-CCGGATCCGTTGATGGGATTTCTAAAAC-3’
r*Hp1489*Sal1	5’-ATGCGTCGACTTAATAAACAAATTCATAAAATAA-3’
r*cagH* Sal1	5’-ATGCGTCGACTCACTTCACGATTATTTTAGTTTG-3’
f*cat*XhoI	5’-CCGCTCGAGTTGTTGATGGGGCAGGCA-3’
r*cat*BamHI	5’-GCGGATCCTTATTTATCTCTGACAAGAG-3’
f*cagI*BamHI	5’-GGGGATCCGTCGACTCTAAAGTTATTGTCAAAT-3’
r*cagI*XhoI	5’-ACCGCTCGAGTAAAAAACATTTCACATCT-3’
f*cag8*BNotI	5’-GGTGGCGGCCGCCAATGAGTGTATTATTTC-3’
r*cag8*BKpnI	5’-GCCCGGTACCATAATAAAGCAACGGATC-3’

Alphabets ‘f’ and ‘r’ indicate forward and reverse primers used to construct plasmids.

### Construction of mutator plasmids and transformation

For creation of *cagI* null mutant strain, mutator plasmid pBS-*cag*8B∆*cagI/CatGC* was constructed following published protocol [7]. Briefly, genomic region of *cag*8B (*cagL, cagI, cagH, cagG* and *cagF* ORFs) sequence was PCR amplified from *H. pylori* 26695 genomic DNA using f*cag*8BN/r*cag*8BK (N & K = NotI and KpnI; f & r = forward and reverse primers respectively) primer pair by *Pfu* polymerase and cloned into pBluescript between KpnI and NotI sites resulting in pBS*cag*8B (**Table S2 **in File **S1** and Table **1**) [7]. Next, plasmid pBS*cag*8B was copied excluding sequence encoding *cag*I by inverse PCR using f*cagI*B/r*cagI*X (B & X = BamHI and XhoI respectively) primer pairs. The inversely amplified PCR product was digested with BamHI and XhoI and ligated with the terminator less *CatGC* cassette amplified from pBS-*CAT* plasmid by PCR using F*cat*X/R*catIB* (X & B = XhoI and BamHI respectively) primer pairs. *E. coli DH5α* competent cells, transformed with the ligated product were platted on LB-agar containing ampicillin. Positive clones were first selected on chloramphenicol plate and finally by double digestion of plasmids isolated from chloramphenicol resistant colonies. Plasmid isolated from positive clone was introduced into *H. pylori* 26695 by natural transformation as described by Haas et al., 1993 and tested for chloramphenicol resistance [38]. Absence of the protein in knock out *H. pylori* strain Hp∆cagI was tested by Western blotting (**Figure S2 **in File **S1**).

### Ethics statement

This study was approved by the Institutional Animal Ethics Committee-of Jawaharlal Nehru University. The Institutional Ethics Committee Code no: 23/2007 and 22/2012.

The animals (Balb/c mice female or New Zealand white rabbit female) were maintained at Central Animal Facility of the Jawaharlal Nehru University as approved by the Institutional Animal Ethics Committee. After experimental procedures were finished, the animals were maintained until their natural death, and every effort was made to minimize their suffering.

### Antibodies, SDS-PAGE and Western blotting

Several polyclonal antibodies against *cag*-PAI proteins were raised in both rabbit and mice in the laboratory. Specificity and titre of raised antibodies such as anti-CagI rabbit antibody, anti-CagH mice antibody, anti-CagX rabbit antibody, anti-CagZ rabbit antibody, anti-CagF mice antibody and anti-CagT mice were tested by Western blotting (**Figure S1 **in File **S1**). Briefly, to generate polyclonal antibodies against CagI and CagH in rabbit and mice respectively, partially purified recombinant CagI (without tag) and His-CagH were used for immunization. *cagI* was cloned in pET-28a vector and expressed without tag in *E. coli* strain BL-21 (DE3). The sarcosine solubilized protein was purified by ion-exchange chromatography. Partially purified CagI protein band was excised from SDS-PAGE gel and used for antibody generation. Likewise His-CagH was purified by affinity chromatography on Ni-NTA agarose beads. The protein bound beads were washed with binding buffer (50mM Tris-HCl, pH 7.4, 150mM NaCl, 1mM EDTA, 1mM PMSF and 5% glycerol) and finally eluted with elution buffer (200mM Imidazole buffer). The pooled fraction containing purified His-CagH was used to raise the mice CagH antiserum.

Rabbit anti-CagI (α-CagI), mice anti-CagH (α-CagH) and rabbit anti-CagA (Santa Cruz, USA) antibodies were used at a 1:10,000, 1:4000, 1:10,000 dilutions respectively. HRP conjugated anti-Biotin antibody (rabbit, Sigma) was used to test biotin labeled protein for characterization.

SDS-PAGE and Western blotting were performed as described previously [7]. Western blotting was performed by transferring protein to PVDF membrane, blocking with 5% Bloto (Genotech Inc, USA) in TBST (50 mM Tris/HCl, pH 7.5, 150 mM NaCl, 0.1% Tween-20) and incubating with the respective antibodies. Horse radish peroxidase conjugated anti-rabbit IgG was used to visualize bound antibody. For visualization of proteins after SDS-PAGE, gels were stained with Coomassie brilliant blue R250 or with silver stain [39].

### Sub-cellular Fractionation of H. pylori

*H. pylori* cells (26695) grown on BHI-serum agar plate were collected and washed with PBS twice and re-suspended in 500µl of 20mM Tris-HCl, pH 8.0. Cell fractionation was performed as described by Doig and Trust, Painbeni et al., and Ge et al., with some modifications [40–42]. Briefly, re-suspended cells were sonicated on ice; unbroken cells and debris were removed by centrifugation at 8000 X g for 10 min at 4^o^C. Cell extract was centrifuged at 140,000 X g for 1 h at 4^o^C in SW-55 rotor Beckman coulter ultracentrifuge. The supernatant fraction was considered as a mixture of cytoplasm/periplasm fractions (S) and pellets were considered as total membrane fraction (TM). Fractionated samples were dissolved in 2X-SDS sample buffer, boiled and subjected to SDS-PAGE, followed by Western blotting using appropriate antibodies.

### Osmotic shock

Freshly grown *H. pylori* cells were harvested gently from BHI-serum agar plate and washed twice with PBS. Cell pellet was re-suspended in buffer having 20mM phosphate buffer, pH 8.0, 20% sucrose, 0.1 mM EDTA and incubated at RT for 10 min. Following centrifugation, cell pellet was rapidly re-suspended (gently) in 100 volume of MQ water containing 0.5mM MgCl_2_ and incubated at RT for 10 min. Thereafter cell suspension was centrifuge at 5,000 rpm for 1 min and supernatant was collected as periplasmic fraction (Os). Residual cell pellet was re-suspended in PBS containing 0.5mM EDTA, lysed by sonication, centrifuged at 1,40000 X g for 1 h at 4^o^C, supernatant and cell pellet were recovered. The supernatant fraction was considered to be cytosolic content whereas residual cell pellet was considered to be total membrane (TM). Isolated fractions were subjected to SDS-PAGE, followed by Western blotting using appropriate antibodies.

### Biotinylation of H. pylori

Intact bacterial cells or whole cell extract were biotinylated following published procedure with minor modifications [26]. For selective biotinylation of periplasmic proteins, freshly grown cells were treated with biotin reagent [Iodoacetate LC-Biotin (Genotech, USA), stock solution (10mg/ml in DMSO)] at a final concentration of 50µg/ml in presence of EDTA to increase the permeability of the outer membrane. This compound reacts preferentially with surface proteins but in the presence of EDTA the reagent can enter into periplasmic space possibly through water filled channels of the bacterial outer membrane porins, resulting in substantial labeling of periplasmic proteins as reported by Sabarth et al., 2002. However, the reagent cannot cross the bacterial inner membrane barrier. Cells were incubated for 30 min on ice. Reaction was stopped by adding 50mM Tris.HCl, 100mM NaCl, 1mM CaCl_2_, and 27mM KCl. After 10 min un-reacted biotin was removed by centrifugation. Biotinylated sample was mixed with SDS loading dye and subjected to SDS-PAGE, followed by Western blotting using appropriate antibodies.

### Immunofluorescence staining and microscopy of H. pylori

Immunofluorescence microscopy (IFM) of *H. pylori* cells was performed as reported earlier, with some minor modifications [43]. *H. pylori* cells were fixed on sterile glass cover slips with 4% paraformaldehyde for 10 min at RT. Following fixation, cells were permeabilized with 0.2% Triton X 100 and cover slides were blocked in 5% bovine serum albumin (BSA) in 1X PBS for 30 min. The cells were then incubated with specific polyclonal antibody of appropriate dilutions (α-CagI-1:1000, α-CagF-1:500, α-CagT-1:1000, α-CagX-1:1000, and α-CagA-1:1000) and respective pre-immune serum (negative control) at 4^°^C for 2 h. Thereafter fixed cells were washed with PBS three times and were further incubated with Cy3-conjugated goat anti rabbit IgG, Alexa fluor 488 goat anti rabbit, and Alexa fluor 594 goat anti mice (Molecular probe) for 1 h at RT as required. The cover slips were mounted with 20% glycerol on glass slides and visualized at 100X through a Carl Zeiss fluorescence microscope equipped with oil immersion objectives. Images were captured using an axio cam Hrm digital camera and analyzed by Axio-vision-4.8 software. The images were processed using standard image processing techniques.

### Co-immunoprecipitation

Either recombinant proteins or *H. pylori* extract were used for co-immunoprecipitation assays. *H. pylori* 26695 cells (~ 40µl cell pellet volume) were re-suspended in 400µl of binding buffer (20mM Sodium phosphate buffer pH 7.4, 150mM NaCl, 2mM EDTA, 10% glycerol, and 6µl of 100x-protease inhibitor cocktail containing 2% DDM, lysed by sonication and centrifuged at 10K rpm for 20 min. The sample was pre-cleared by adding pre-immune rabbit serum and protein-A agarose beads. Each pre-cleared sample was divided into two equal parts; 3 µl of pre-immune rabbit serum was added to one part, and 2-3µl primary antibody (3µl anti-CagI antibody) was added to the other part and incubated for 3 h at 4^°^C on a vertical rocker. Following incubation, 15µl of protein-A agarose bead was added to each sample and incubated for additional 1-2 h. Following brief centrifugation at 5000 X g, supernatant was discarded and beads were washed with binding-buffer. Bound protein was released by boiling the beads with SDS-sample buffer, subjected to SDS-PAGE, followed by Western blotting using desired antibody.

### Electron microscopy

Freshly grown *H. pylori* cells were re-suspended in phosphate buffer pH 7.4 to an optical density of approximately 1.0 at 600 nm and were fixed with fixing agent (2% paraformaldehyde plus 2.5% glutraldehyde in 100 mM phosphate buffer, pH 7.0) for 6 h at 4^0^C. Cells were dehydrated by treatment with a graded ethanol sequentially, embedded in L R White 450 resin at 60^0^C for 48 h. Ultrathin sections (80–100 nm) were cut and placed on copper TEM grids. After blocking, the grids were incubated with the primary antibodies (α-CagI, and α-CagA at a dilution of 1:100) at 4^0^C in a humidified chamber for 2 h followed by 1 h incubation with Protein-A conjugated to colloidal gold particles (15 nm in size, EY laboratories). The grids were negatively stained with 4% phospho-tungstate uranyl acetate (pH 4.0), and examined in a JEM-2100F (JEOL) transmission electron microscope.

## Supporting Information

File S1 (DOC)
