# Supplementary material for: Cag Type IV Secretion System: CagI Independent Bacterial Surface Localization of CagA
Source: PLoS One. 2013 Sep 10;8(9):e74620. doi: 10.1371/journal.pone.0074620 (PMC3769253; doi:10.1371/journal.pone.0074620)
Supplement: File S1 — (DOC) [file pone.0074620.s001.doc]

**SUPPLEMENTARY TABLES AND FIGURES**

**Table SI: *H. pylori* and *E. coli* strains used in the present study.**

| **Strains** | **Descriptions** | **References** |
| --- | --- | --- |
| *H. pylori 26695* | *Helicobacter pylori* wild type strain | Tomb *et. al*., 1997 |
| *Hp26695∆cagZ* | Deletion mutant for *hp0526* (*cagZ*) | This study |
| *Hp26695∆cagV* | Deletion mutant for *hp0530* (*cagV*) | This study |
| *Hp26695∆cagT* | Deletion mutant for *hp0532* (*cagT*) | Fischer *et. al*., 2001. |
| *Hp26695*∆*cagX* | Deletion mutant for *hp0528* (*cagX*) | This study |
| *Hp26695∆cagI* | Deletion mutant for *hp0540* (*cagI*) | This study |
| *Hp 26695*∆*cagA* | Deletion mutant for *hp0547* (*cagA*) | Fischer *et. al*., 2001 |
| *Hp26695∆cagM* | Deletion mutant for *hp0537* (*cagM*) | This study |
| *Hp26695∆cagY* | Deletion mutant for *hp0527* (*cagY*) | This study |
| *E. coli BL21(DE3)* | Host for co-expression | Novagen |
| *E. coli DH5α* | General cloning host | Berlyn MKB, 1996 |

**Table S2: List of set of primer pairs used for amplification of gene fragment for construction of recombinant plasmids.**

| **construct name** | **primer pair used for amplification** | **cloning site** |
| --- | --- | --- |
| pET-*cagI*A | F*cagI*N/R*cagI*H | NcoI/HindIII |
| pACYC-*cagI*A | F*cagI*N/R*cagI*H | NcoI/HindIII |
| pGEX-*Hp1489* | F*Hp1489*B/R*Hp1489*S | BamHI/SalI |
| pMAL-*cagH* | F*cagH*B/R*cagH*S | BamHI/SalI |
| pBS-*cag*8B∆*cagI/CatGc* | f*cagI*B/r*cagI*X | BamHI/XhoI |
| pBS-*cag*8B | f*cag*8BN/r*ag*8BK | KpnI/NotI |

**Figure S1. Polyclonal antibodies raised against *cag*-PAI proteins in rabbit and mice***. Wild type H. pylori 26695, mutant strains and recombinant protein cell extract were used to check specificity and titre of polyclonal antibodies by Western blotting as indicated****. (A)*** *Polyclonal anti-CagI rabbit antibody,* ***(B)*** *anti-CagH mice antibody,* ***(C)*** *anti-CagX rabbit antibody,* ***(D)*** *anti-CagZ rabbit antibody,* ***(E)*** *anti-CagF mice antibody and* ***(F)*** *anti-CagT mice antibody as indicted. Pre-immune serum (preimm.), antibody dilutions and molecular weight marker of the proteins are indicated.*

**Figure S2. Western blots showing absence of CagI in *Hp*Δ*cagI.*** *Equal amount of wild type H. pylori (Hp) and* *Hp*Δ*cagI total cell extract were loaded. CagX used for loading control. Antibodies used are marked.*

**Figure S3: Western blots showing cellular fractionation of wild-type *H. pylori* and *Hp*Δ*cagI*.** *Cellular localization of CagA in wild type H. pylori (Hp) and Hp*Δ*cagI. TC, S and TM indicate total-cell lysate, soluble (cytoplasmic/periplasmic) and total membrane fractions respectively. CagX and CagT are membrane associated proteins used as positive control. Antibodies used are marked.*

**Figure S4: CagI is essential for CagA translocation into AGS cells*.*** *AGS, human gastric epithelial cells*, *were grown in the presence of 5% CO_2_ in RPMI medium containing 10% FBS. AGS cells were infected with* H. pylori (Hp)*and Hp*Δ*cagI strains at a multiplicity of infection of 1:100 for 4 hours. Infected cells were washed with PBS pH-7.4 twice. AGS cells infected with wild type H. pylori are showing cell elongation, however, Hp*Δ*cagI were failed to do so indicating there is no translocation of CagA into host cells.*

**Figure S5: Cag-T4SS genes including *cagI* are required for pili formation**. *H. pylori and cag-PAI mutant strains were grown on solid Brain heart infusion agar plate as described in materials and methods. AGS cells were co-cultured with wild type H. pylori and its isogenic mutant strains with MOI of 1:100 for 5 hours at 37^0^C. Bacterial cells were fixed in 2.5% glutaraldehyde and subsequently dehydrated with graded ethanol. Dehydrated cells were chemically dried with HMDS (1,1,1,3,3,3,Hexamethyldirilazone), mounted onto sample stubs, grounded with silver paint at the sample edge and sputter-coated with palladium-gold before viewing with an Carl Zeiss Evo40 scanning electron microscope. (****A****) wild-type H. pylori (Hp). (****B****) Hp*Δ*cagI. (****C****) Hp*Δ*cagV. (****D****) Hp*Δ*cagT. (****E****) Hp∆cagδ. White arrows indicate T4SS pili structures. Scale bars indicate 200 µM.*

**Figure S6. IFM showing analysis of CagA localization in *Hp∆cagT* and *Hp∆cagM* mutant strains.** *H. pylori cells were fixed and permeabilized with 0.2% TritonX-100 as described in Materials and Methods. NP and P stand for non-permeabilized and permebilized cells respectively. Primary antibodies used in IFM are indicated. Cy3 (red colour) conjugated secondary antibody was used for final detection by immunofluorescence microscopy.*
